# Features of *Blastocystis* spp. in xenic culture revealed by deconvolutional microscopy

**DOI:** 10.1007/s00436-015-4540-x

**Published:** 2015-05-22

**Authors:** Robyn Nagel, Christian Gray, Helle Bielefeldt-Ohmann, Rebecca J. Traub

**Affiliations:** School of Veterinary Science, University of Queensland, Gatton, Queensland Australia; Plymouth University Peninsula Schools of Medicine and Dentistry, Plymouth University, Plymouth, UK; Australian Infectious Diseases Research Centre, University of Queensland, St Lucia, Queensland Australia; Faculty of Veterinary and Agricultural Sciences, University of Melbourne, Parkville, Victoria Australia

**Keywords:** *Blastocystis*, Deconvolutional microscopy, Autofluorescence

## Abstract

*Blastocystis* spp. are common human enteric parasites with complex morphology and have been reported to cause irritable bowel syndrome (IBS). Deconvolutional microscopy with time-lapse imaging and fluorescent spectroscopy of xenic cultures of *Blastocystis* spp. from stool samples of IBS patients and from asymptomatic, healthy pigs allowed observations of living organisms in their natural microbial environment. *Blastocystis* organisms of the vacuolated, granular, amoebic and cystic forms were observed to autofluorescence in the 557/576 emission spectra. Autofluorescence could be distinguished from fluorescein-conjugated *Blastocystis*-specific antibody labelling in vacuolated and granular forms. This antibody labelled *Blastocystis* subtypes 1, 3 and 4 but not 5. Surface pores of 1 μm in diameter were observed cyclically opening and closing over 24 h. Vacuolated forms extruded a viscous material from a single surface point with coincident deflation that may demonstrate osmoregulation. Tear-shaped granules were observed exiting from the surface of an amoebic form, but their origin and identity remain unknown.

**Electronic supplementary material:**

The online version of this article (doi:10.1007/s00436-015-4540-x) contains supplementary material, which is available to authorized users.

## Introduction

*Blastocystis* spp. are common enteric, unicellular parasites found in almost every species of animal (Tan 2008). Seventeen different subtypes (STs) defined by the 18SSU of the ribosomal RNA gene are recognised, and ST3 is almost universally the most common of the nine STs found in humans (Stensvold 2012). *Blastocystis* infection has been linked to irritable bowel syndrome-like symptoms in humans, but epidemiological studies are inconclusive (Scanlan 2012). Many healthy people carry *Blastocystis* sp., and it remains unclear if the parasite is pathogenic. Pathogenicity has been speculated to relate to parasite subtypes (Eroglu and Koltas 2010; Tan et al. 2006) as well as to the host’s immune response (Olivo-Diaz et al. 2012).

Analysis of the genome of two different STs of *Blastocystis* sp. has allowed prediction of genes and proteins (Denoeud et al. 2011) that may be associated with the organism’s pathogenic potential. Nevertheless, a lack of knowledge of the basic life cycle and metabolic function of the parasite remains a major limitation to exploiting the use of animal models and in vitro systems to further explore the clinical significance and therapeutic options for the control of this organism.

**Fig. 1:**
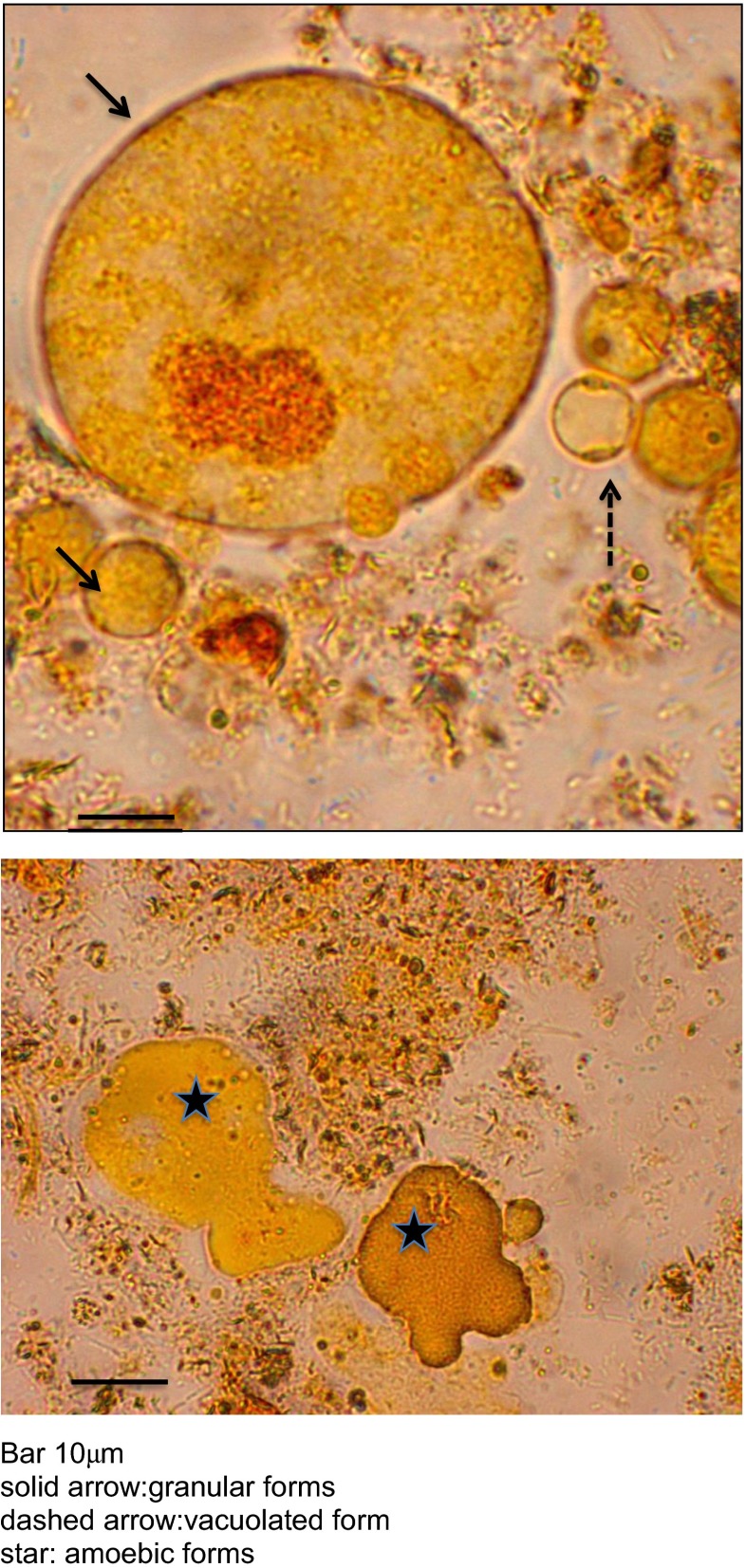
Morphological forms of *Blastocystis* sp. in xenic culture stained with acridine orange

Studies utilising light microscopy, transmission (TEM), scanning (SEM) and freeze-etch (FE-EM) electron microscopy have described multiple forms of the *Blastocystis* organism, including vacuolated (VF), granular (GF), amoebic (AF) and cystic (CF) forms (Fig. 1). The relationship of these different forms to each other is unclear (Tan 2008), although it is certain that the robust cystic form transmits infection (Moe et al. 1997). Microscopic images have often been obtained from attenuated or axenic cultures. These elegant studies have been useful in describing the intricate ultrastructure and surface morphology of the various forms of *Blastocystis* spp., but their limitation is that they capture still images of a dead organism separated from the usual microbial environment. In this study, we employed deconvolutional microscopy of xenic cultures of living *Blastocystis* sp. to obtain time-lapse and three-dimensional images of the *Blastocystis* organisms. This microscope also has the capability to record fluorescence in various light spectra facilitating utilisation of *Blastocystis*-specific fluorescent antibodies and fluorescent double-stranded deoxyribonucleic acid (DNA) stain, 4′,6-diamidino-2-phenylindole (DAPI) (Sigma-Aldrich, Australia).

## Materials and methods

### Sample preparation

Fresh faecal specimens were obtained from irritable bowel syndrome patients positive for *Blastocystis* carriage and from pigs at the University of Queensland (UQ) Gatton Campus piggery, obtained in accordance with University of Queensland Medical and Animal Ethics Committee approvals numbers 2011000454 and 2012000069, respectively. Ten grams of faeces was subcultured aerobically for 24–48 h in Jones medium (Jones 1946) supplemented with 10 % heat-inactivated horse serum. The sediment at the interface between the basal residue in the test tube and the liquid medium was removed for microscopic analysis.

### Slide preparation

Fifteen microliters of fresh sediment was mixed gently with 10 μL phosphate-buffered saline and placed on a glass slide. The edges of the slide cover were sealed to prevent air entry to the specimen. An inverted slide was placed into a Deltavision Elite deconvolution microscope (Applied Precision, GE Healthcare, Berthold Australia, Bundoora, Victoria, Australia) and examined with polarised light and with fluorescent filters. Four different filters that allowed excitation and emission ranges from 350 to 700 nm, including DAPI, fluorescein isothiocyanate (FITC), tetramethylrhodamine (TRITC) and cyanine 5 (Cy5) spectroscopy ranges were used for examination. The images were magnified 20 times or 40–60 times with oil immersion and repeated images were taken at cross sections through the specimen or at different time intervals. Time-lapse microscopy was performed over 24 h with images taken every 15 min. The environmental chamber was maintained at 37 °C throughout the microscopy session.

### Antibody staining

FITC-conjugated *Blastocystis*-*s*pecific polyclonal antibody raised against axenic ST3 (Blastofluor®; Antibodies Inc., David, CA, USA) was also used to assess the specimens. Two hundred microliters of sediment was incubated with 5 μL of Blastofluor® for 30 min at 37 °C. Staining of double-stranded DNA with DAPI was performed by adding 2 μL of stock solution of DAPI (0.5 μg DAPI in 1 mL PBS) to the prepared microscopy slide and gently mixed before incubating in the dark for 10 min.

## Results and discussion

### Auto- and immunofluorescence

**Fig. 2:**
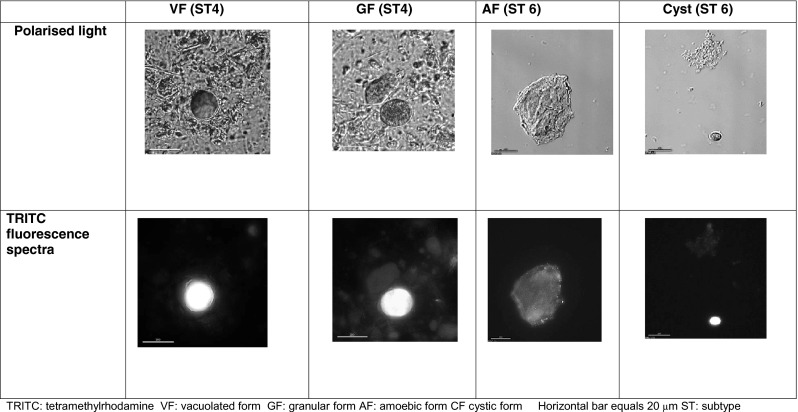
Autofluorescence of different morphological forms of *Blastocystis* spp.

**Fig. 3:**
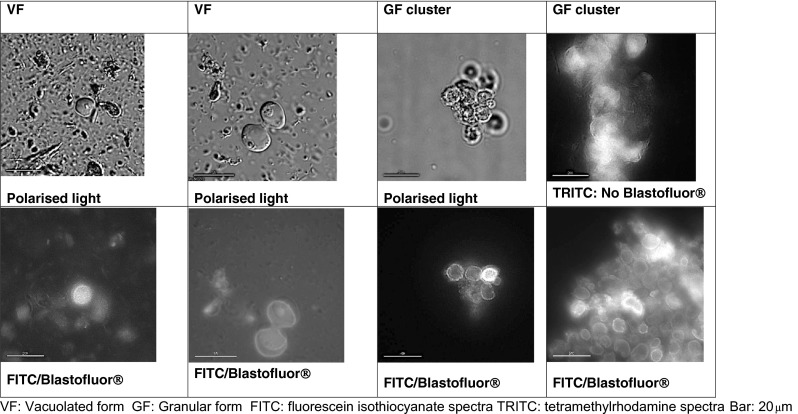
Different morphological forms of *Blastocystis* spp. stained with Blastofluor®

Autofluorescence was observed in VF, GF and AF and the CF (Fig. 2) of *Blastocystis* cells. The autofluorescence was greatest in the TRITC excitation/emission light spectra (557/576-nM) and appeared to be brightest in the central vacuole, with spots of light emission observed at the surface of the organism. The autofluorescence faded with time and appeared to be optimal in fresh VF forms, CF and least in AF. The autofluorescence was distracting but could be distinguished from the Blastofluor® stain in the VF and GF. The Blastofluor® stained the external cell membrane of VF, GF but not the AF (Fig. 3) and was brightest using the FITC (495/515-nm) light spectrum. The Blastofluor® stain could not be reliably differentiated from the strong autofluorescence seen in the CF. The Blastofluor® antibody stained the VF and GF of STs 1, 3 and 4 specimens but did not stain these forms in ST5 pig samples. DAPI staining of VF stained the nuclei on opposite poles of the central vacuole a dense bright blue.

Non-specific green autofluorescence (GAF) was originally thought to be a rare occurrence in microalgae that might prove a useful taxonomic feature. However, over time, GAF has been identified in many different organisms including algae, dinoflagellates, diatoms, cyanobacteria and raphidophytes (Tang and Dobbs 2007). The GAF occurs in the cytoplasm of these microalgae as well as in cysts that have little cytoplasm and is affected by various types of stress on the cell such as light, heat and drying and is caused by molecules other than chlorophyll. The chemical nature of GAF is unknown but is likely caused by multiple compounds or multiple derivatives of a single compound, such as flavin-like molecules, or luciferin compounds (Tang and Dobbs 2007). A variety of stains that fluoresce in the green light spectrum are used to assess morphology and physiological states of organisms and GAF has been reported to interfere with the interpretation of these stains (Tang and Dobbs 2007). This is the first report of autofluorescence by *Blastocystis* organisms. *Blastocystis* diverged in evolution at the time of red algae and the genome suggests incorporation of cyanobacteria genes (Denoeud et al. 2011), so the presence of GAF is not unexpected. The GAF was seen strongly in the central vacuole of the VF. Little is known of the chemical constituents of this vacuole except that it stains positively for carbohydrates (Yoshikawa et al. 1995a) and lipids (Yoshikawa et al. 1995b).

The GAF did not interfere with the ability to detect Blastofluor® staining at the surface of the organism in the VF and GFs but could not be differentiated from GAF in cysts. *Blastocystis* cysts are difficult to identify in stools, but as GAF is a relatively non-specific finding in microalgae, screening with fluorescent filters may not aid detection of the CF. Blastofluor® did not stain the AF of *Blastocystis* suggesting that the surface antigens of this form differ from the VF and GF. Blastofluor® is a polyclonal antibody raised in rabbits to axenic cultures of ST3 and has been shown to react to *Blastocystis* organisms with no cross reactivity with *Giardia duodenalis*, *Entamoebae histolytica* or *Cryptosporidium* spp. (Dogruman-Al et al. 2010). *Blastocystis* immunolabelling with Blastofluor® has previously been shown to stain faecal samples of steers, goats and humans, but not pigs after prolonged (up to a year) storage in formalin (Gould and Boorom 2013). This is consistent with our own observations in which no Blastofluor® staining was detected in our ST5 pig samples. ST5 organisms are commonly detected in pigs worldwide and locally, 100 % pigs have previously been shown to carry *Blastocystis* sp. ST5, and 7 % harbour mixed infection with ST1 (Wang et al. 2014). This suggests that the surface antigens display homology between some STs (ST 1, 3, 4) but that ST5 organisms differ in some respect to some surface antigens.

### Cell granules and time-lapse microscopy

**Fig. 4:**
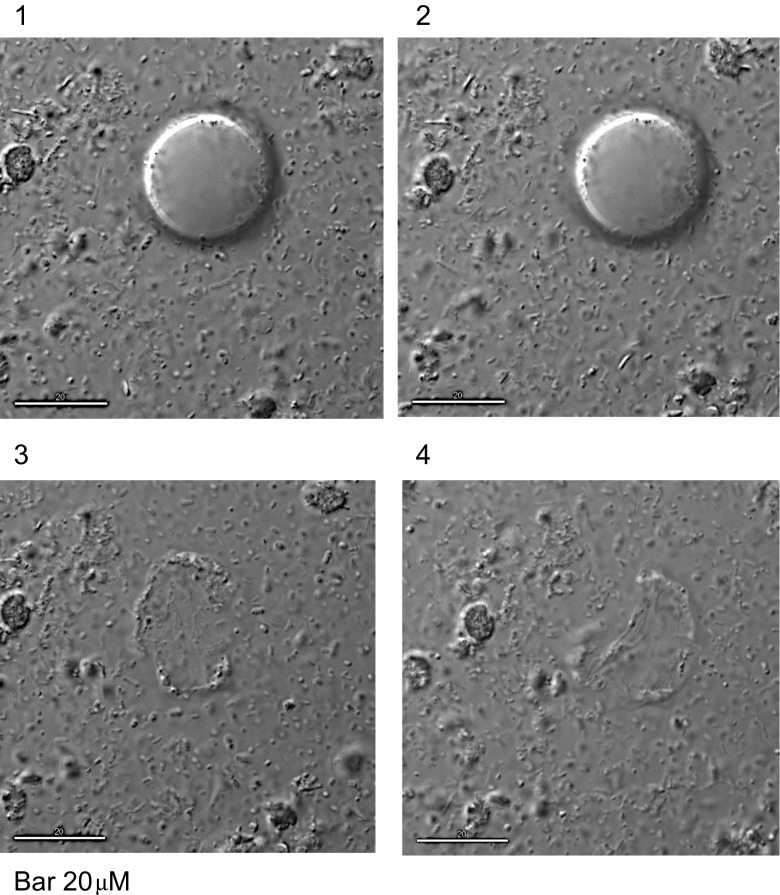
Vacuolated form of *Blastocystis* sp., 15-min time-lapse sequence

**Fig. 5:**
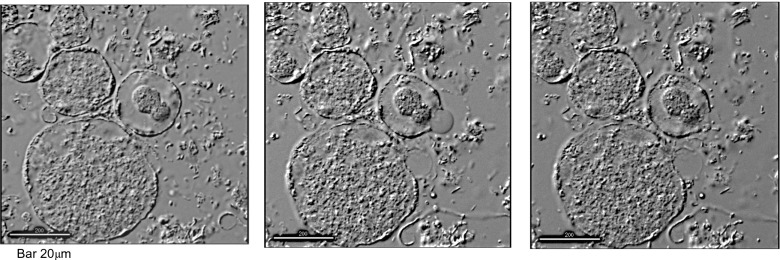
Xenic culture of *Blastocystis* granular forms, time-lapse images over 24 h

**Fig. 6:**
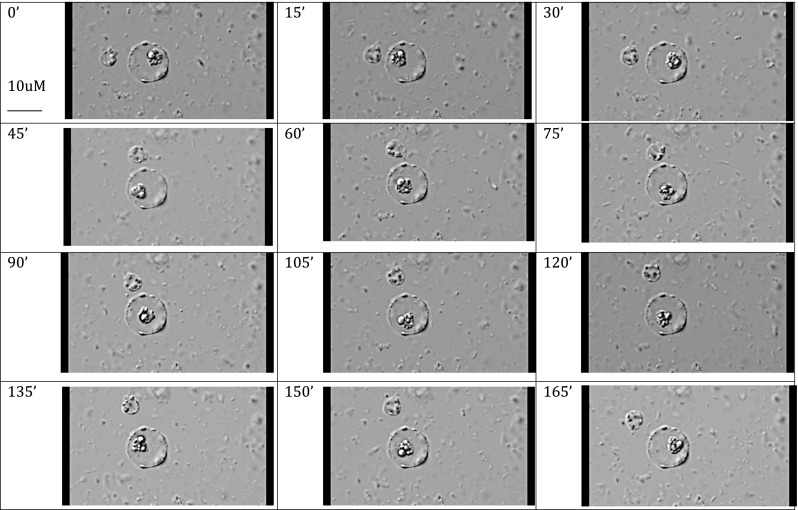
*Blastocystis* with moving central granule sequential images over 15-min time intervals

**Fig. 7:**
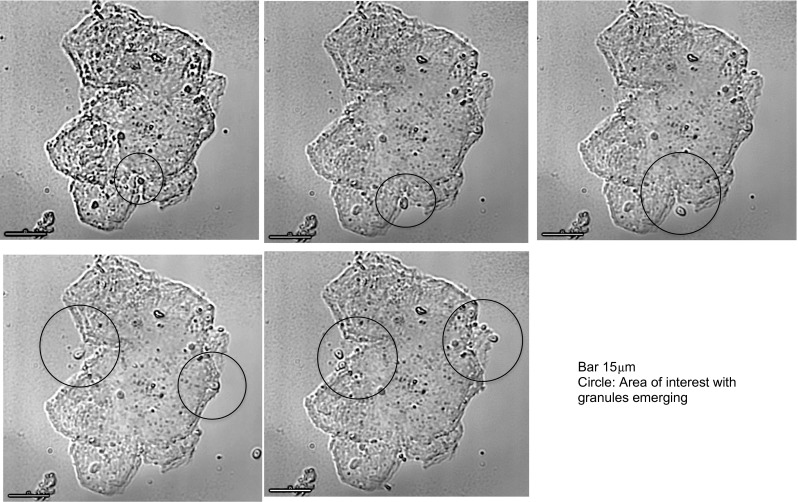
Granules exiting *Blastocystis* amoebic form in xenic culture, time-lapse images taken over 24 h

Detailed views of *Blastocystis* organisms were obtained, but apart from observing binary fission developing in vacuolated and granular forms, we did not observe changes from one morphological form to another. It was difficult to keep the *Blastocystis* organisms in the field of view despite using the cell tracking facility of the microscope. The *Blastocystis* cells were fragile and would often either rupture (Fig. 4) or drift away out of the field of view over the 24 h of recording, preventing observation of progressive cell changes past a certain point (Fig. 5). One GF, observed over 6 h in total, that appeared stationary on the slide displayed a large central granule moving rapidly within the GF (Fig. 6, Supplementary video file 1). Another time-lapse study of a granular AF showed the form enlarging and losing granularity on the surface with subsequent expulsion of small tear-shaped granules. These granules appeared to arise from the surface of the organism or track along fissures to the surface (Fig. 7). These granules varied in size from 1 to 3 μm and appeared to have a central dark spot surrounded by clear cytoplasm with a thick dark surface membrane. The release of the granules from the surface was best appreciated in the video images (Supplementary video file 2). Granules that appear to have tracked along fissures are identified as an area of interest in Fig. 7.

The life cycle of *Blastocystis* spp. remains elusive and the function of the multiple inclusion granules present in the GF is still unknown. These granules have been proposed to have functions related to metabolism, storage or reproduction (Zierdt 1991). DAPI has been shown to stain VF double-stranded DNA strongly in the nucleus and weakly in the mitochondria-like organelles (Stenzel and Boreham 1996) but did not stain the central vacuole or the granules of GFs (Matsumoto et al. 1987). These granules have been classified into three different types based on TEM studies (Tan and Zierdt 1973), namely myelin-like inclusions, crystalline granules and lipid droplets (Dunn et al. 1989). None of these previous microscopy studies have supported the hypothesis that these central granules constitute a reproductive phase. The cause of the movement of the large central granule seen within the GF in this study (Fig. 6) is not understood, and simple Brownian motion cannot be excluded.

The small granules that were expelled from the granular AF are intriguing (Fig. 7) but DAPI stains were not performed on the AF to confirm a reproductive role. AF are reported to occur commonly in faeces (Stenzel and Boreham 1996), particularly in patients with symptomatic diarrhoea (Tan and Suresh 2006), but only rarely in culture. Asexual binary fission of VF and GF occur commonly in culture but other routes of reproduction have not been proven (Tan and Stenzel 2003). It is possible that the gut microbiota is necessary for completion of the normal life cycle of *Blastocystis* and that sexual replication may occur only in primed AF in the gut.

### Other surface changes observed over time

**Fig. 8:**
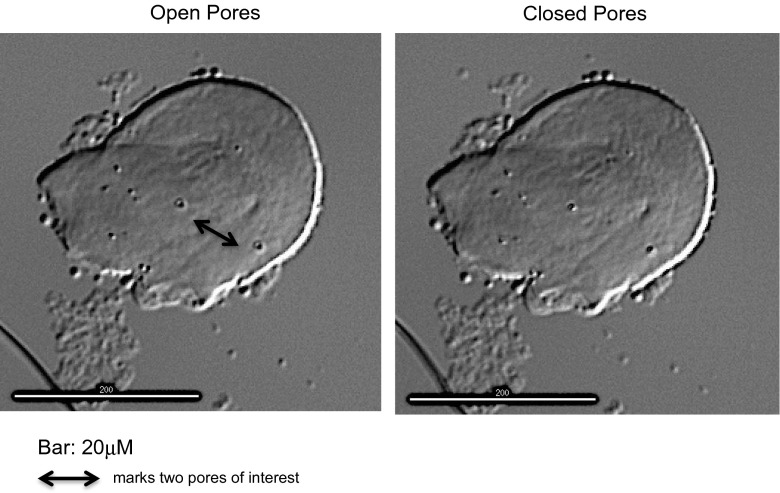
*Blastocystis* form in xenic culture showing pores on surface

Surface pores, with an approximate diameter of 1 μm, were seen on the external surface of the cell membrane of a partially ruptured ST3 *Blastocystis* organism (either a VF or a GF). The time-lapse recording of this event shows a total of 15 pores opening and closing (Supplementary video file 3). They appear to open and close fully over 1 h during four 15-min interval observations. The regular movement of the pores continued for the length of the 24 h study. A time interval sequence is shown with two pores in the foreground demonstrating fully open and closed pore positions in Fig. 8.

The presence of surface pores concurs with previous EM findings. Using TEM, four morphological types of electron dense pits have been described on the surface membrane of the cell wall of *Blastocystis* sp., namely bars, cup-shaped pits, alveolate pits and tubular pits (Dunn et al. 1989). SEM studies have also confirmed the presence of small indentations on the external cell wall membrane, with fewer indentations noted on cells in prolonged culture (Cassidy et al. 1994). FE-EM has shown pores, approximately 0.5 μm in diameter, on the external surface of the outer cytoplasmic membrane that correspond to indentations on the inner surface of the outer cytoplasmic membrane (Tan et al. 1974), suggesting that pores may connect the outer and inner cytoplasmic membrane into the central vacuole. Unfortunately, FE-EM damages the external layer of the outer cytoplasmic membrane so that FE-EM cannot confirm if these pores connect all the way through the cell surface.

**Fig. 9:**
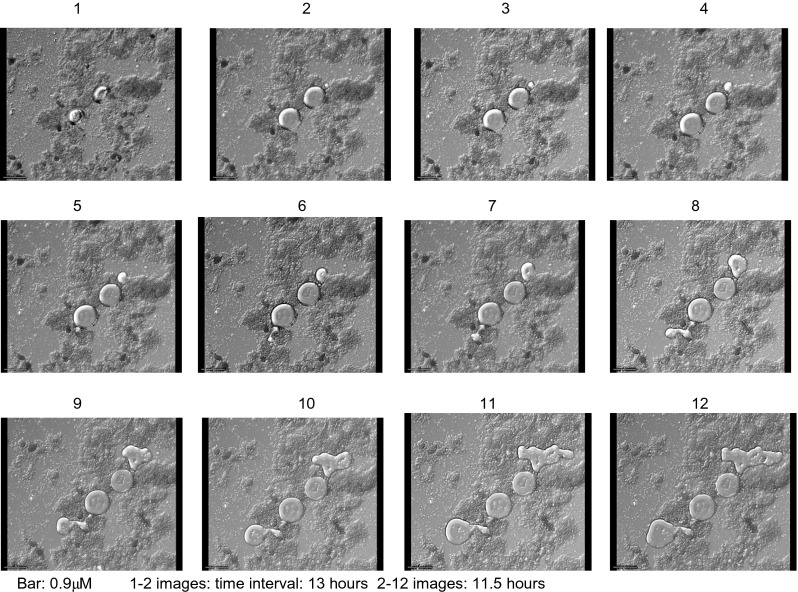
Xenic culture of *Blastocystis* vacuolated form with extrusion, time-lapse images

**Fig. 10:**
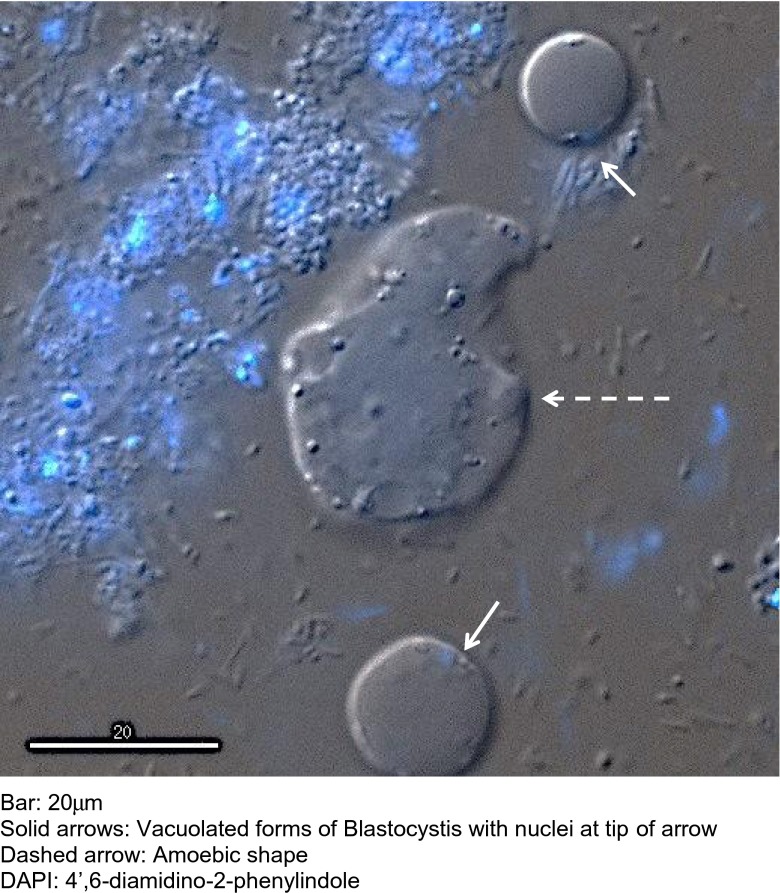
Fluorescent microscopy of *Blastocystis* forms stained with DAPI

Another time-lapse series of images showed extrusion of a viscous material apparently from one single point on the external cell membrane of two VFs of *Blastocystis* (Fig. 9) (Supplementary video file 4). The *Blastocystis* organisms were first seen to increase in size over 13 h then deflate, but without disintegrating, as this substance extruded. This extrusion process took 11.5 h in total, and the extruded material had an amoebic shape. A separate DAPI-stained slide (with multiple images taken at cross sections through the slide, but not time-lapse) stained nuclei in adjacent VFs bright blue, but there was no discrete nuclear material staining seen in the adjacent amoebic shape. This amoebic-shaped structure appeared very similar in shape to the extruded material observed in the previous slide (Fig. 10). The centre of this amoebic structure stained a light diffuse blue, and the surface is covered in granules that do not fluoresce with the DAPI stain.

This curious extrusion of viscous material from the VF occurring over many hours was observed to occur with deflation but conservation of the VF (Fig. 9). Typically, VFs have only a fine rim of cytoplasm surrounding a large central vacuole. The large amount of this viscous material extruded from a single point occurring simultaneously with deflation of the VF is consistent with a direct connection from the surface to the central vacuole. This fluid extrusion may be occurring through the surface pores, although it was interesting to note that the fluid only appeared to exit from one place on the surface wall and not from multiple pores.

The regular, repetitive movement of pores opening and closing in these time-lapse images suggests that the movement is active and requiring energy, even in this disrupted cell. Surface pores have been reported to be important for nutrition and osmoregulation in other protozoa (Bokhari et al. 2008). The possibility that this viscoid extrusion represented the formation of a different morphological form, namely the development of an AF from a VF, was considered. The similar amoeboid shape recorded on a different day showed no evidence of nuclear material within it (Fig. 10) and provided no support for this hypothesis. Over the 24 h of the study, the material on the slide would have depleted nutrients and become progressively dehydrated, and it is possible this fluid extrusion is an osmoregulatory process occurring in a stressed VF. *Blastocystis* spp. have been reported to survive in varying osmotic conditions (Ho et al. 1993), although only cysts survive immersion in fresh water. Osmosensing and osmoregulation have been reported in yeasts, microalgae and protozoa (Suescun-Bolivar and Thome 2015). Protozoans have been reported to osmoregulate variously by changing the concentration of alanine, effluxing ions and water from either contractile vacuoles or intracellular compartments as well as inhibiting mitochondrial function during hyperosmolar conditions (Suescun-Bolivar and Thome 2015).

## Summary

Deconvolutional microscopy of xenic cultures of *Blastocystis* spp. produced images of living organisms in their natural microbial environment but proved challenging due to the fragility of the organisms. Fluorescent spectrometry revealed that *Blastocystis* cells exhibit green autofluorescence in common with many other microalgae. GAF was able to be distinguished from fluorescein-specific *Blastocystis* antibody labelling in VF and GF but not cysts. Care will need to be taken in future studies to distinguish GAF from other stains fluorescing in the green light spectrum. Surface pores were observed and appeared to be opening and closing actively. Extrusion of a viscoid material from VF may be occurring via these pores. Coincident with the extrusion, the VF appeared to deflate, suggesting a direct connection from the surface to the central vacuole. This extrusion may be an osmoregulatory mechanism used by *Blastocystis* spp. to maintain cell homeostasis and integrity. Further studies could use time-lapse images to examine changes in organisms immersed in hypo-osmolar and hyperosmolar medium to further explore this hypothesis. The nature of the tear-shaped granules observed exiting from an amoebic form is unclear. Amoebic forms are common in diarrheal faeces and rarely observed in culture. It is possible these are reproductive granules occurring only in amoebic forms that have been primed by the gut faecal microbiota. Fluorescent nuclear stains to prove transmission of genetic material could be combined in future time-lapse studies of amoebic forms in xenic cultures.

## Electronic supplementary material

Supplementary Video 1 *Blastocystis* with moving central granule sequential images over 15 minute time intervals (M4V 3694 kb)

Supplementary Video 2 Granules exiting *Blastocystis* amoebic form in xenic culture, time-lapse images taken over 24 hours (MP4 10684 kb)

Supplementary Video 3 *Blastocystis* form in xenic culture showing pores on surface (MOV 9221 kb)

Supplementary Video 4 Xenic culture of *Blastocystis* vacuolated form with extrusion (MP4 5422 kb)
